# Supplementary material for: Identification of a Novel Model for Predicting the Prognosis and Immune Response Based on Genes Related to Cuproptosis and Ferroptosis in Ovarian Cancer
Source: Cancers (Basel). 2023 Jan 18;15(3):579. doi: 10.3390/cancers15030579 (PMC9913847; doi:10.3390/cancers15030579)
Supplement: Supplementary file 1 [file cancers-15-00579-s001.zip › Supplementary Figures.pdf]

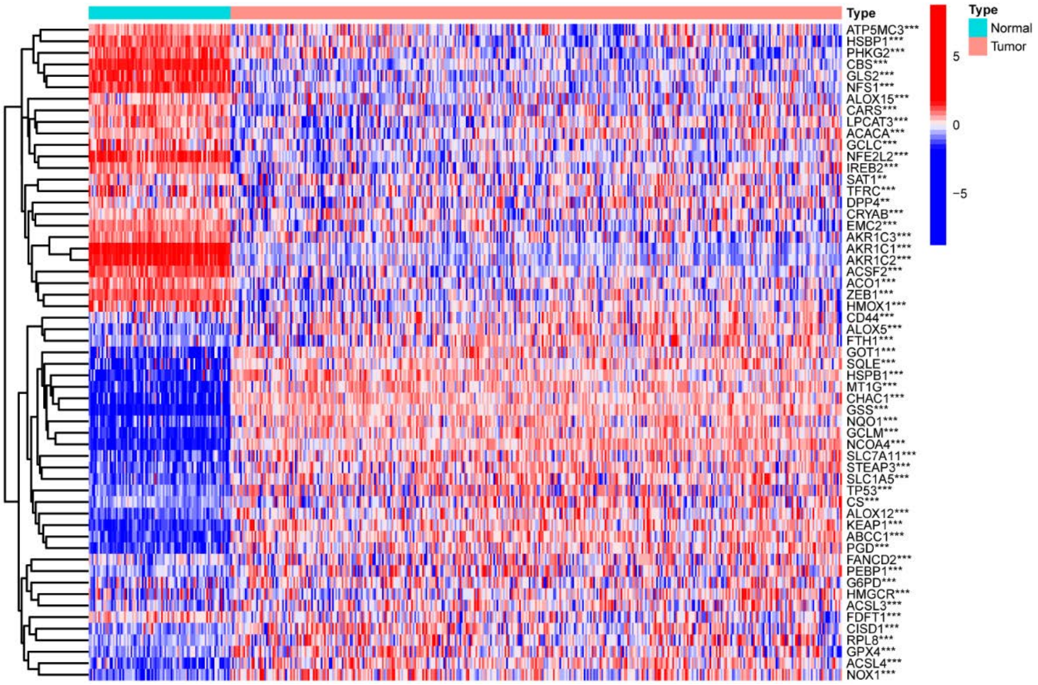

Supplementary Figure S1. Based on TCGA-OV and GTEx-ovary, 57 differentially expressed ferroptosis-related genes were identified.

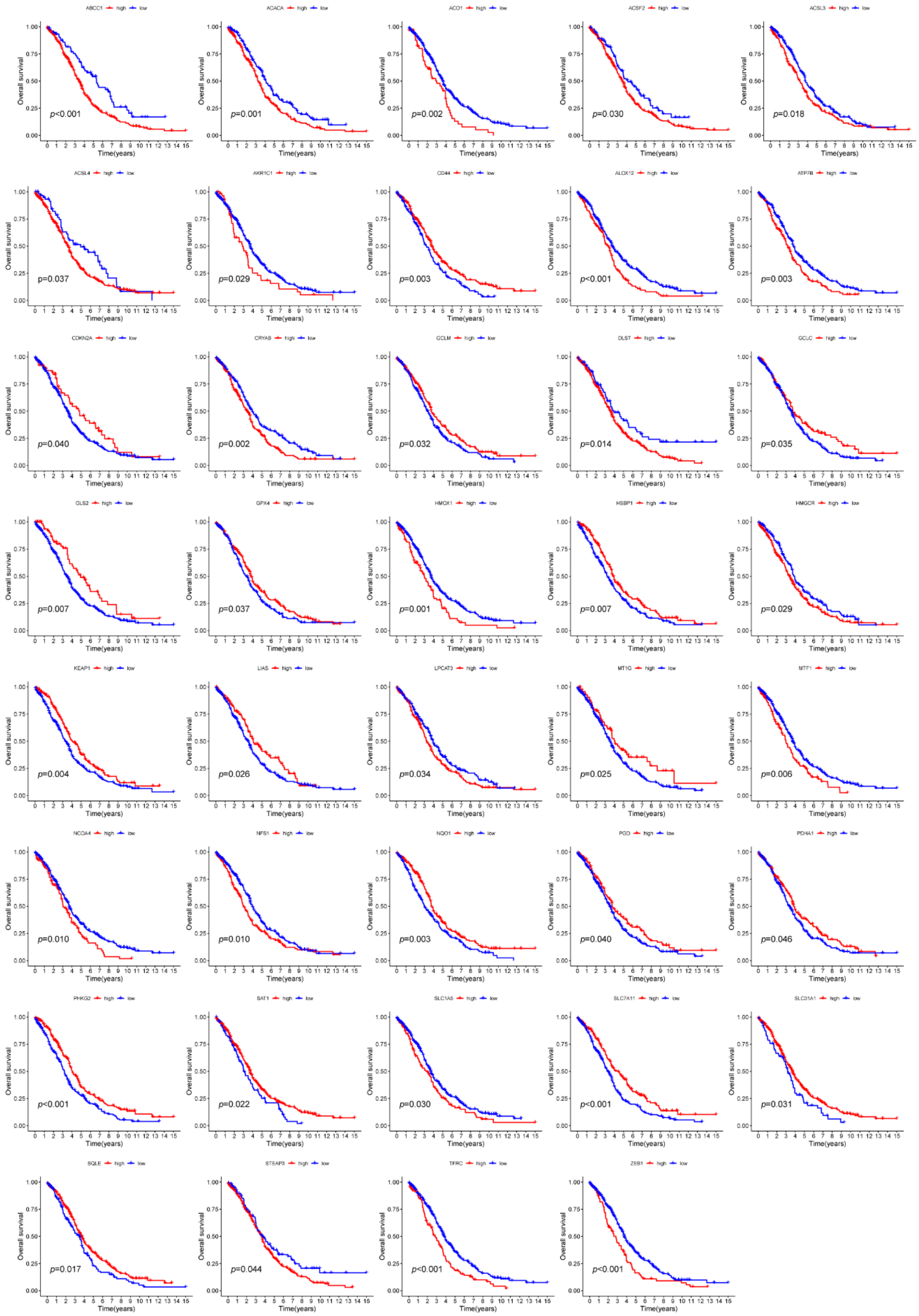

Supplementary Figure S2. 39 prognosis-related genes (*PRGs*) were identified for our subsequent analysis.

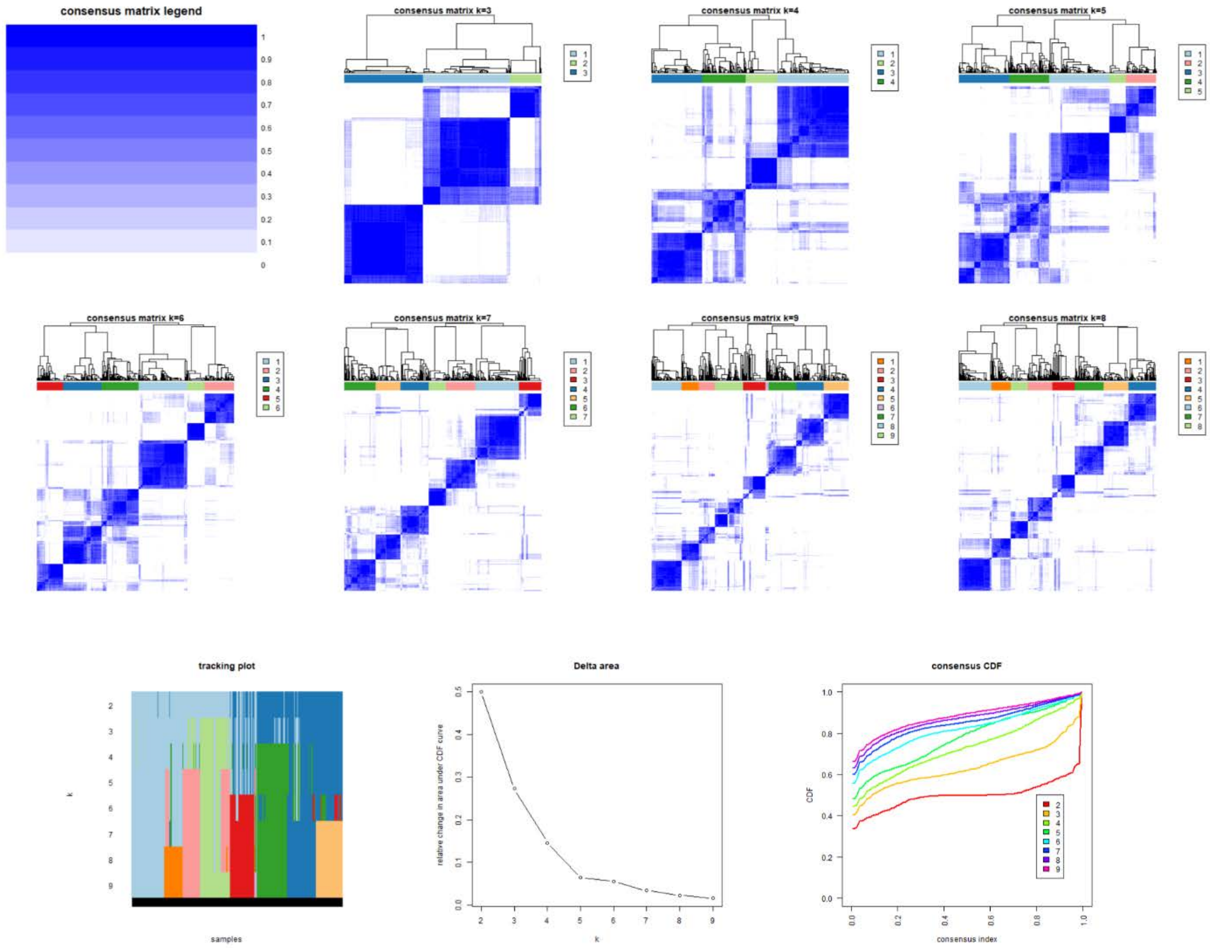

Supplementary Figure S3. Consensus matrixes for all samples were clustered into an appropriate number of prognosis-related clusters (k=1-9).

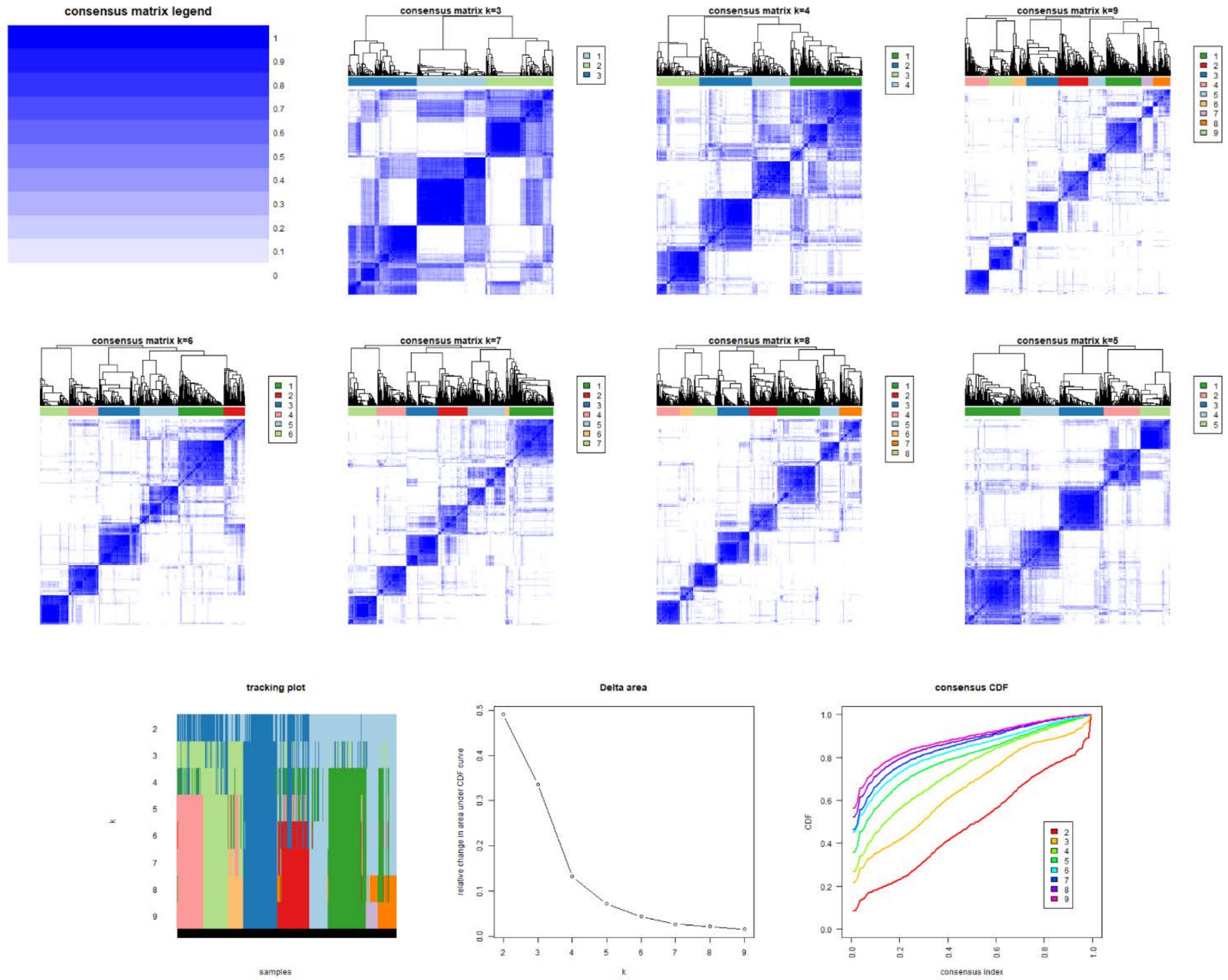

Supplementary Figure S4: Consensus matrix identifies different gene clusters (k = 1–9).

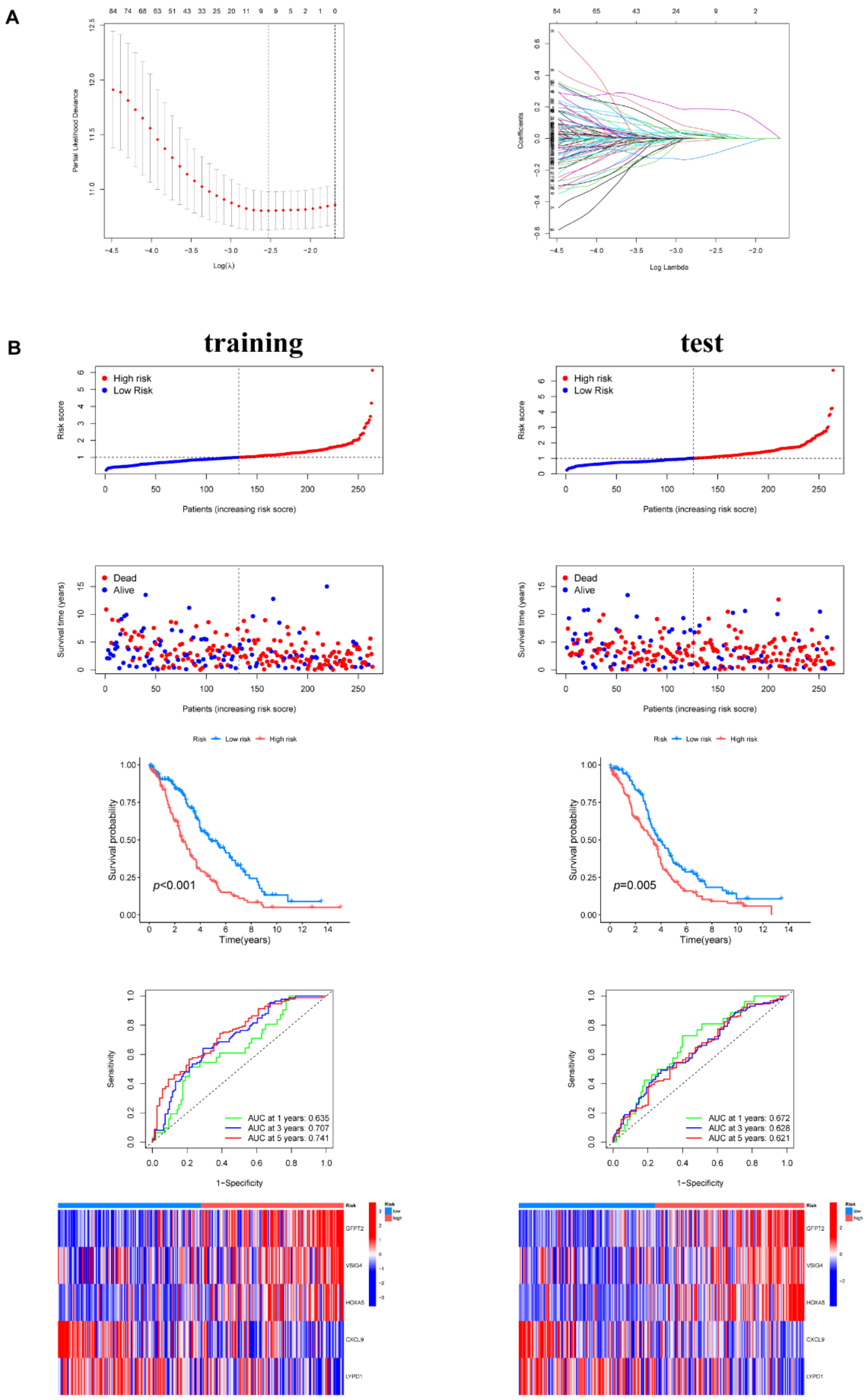

Supplementary Figure S5: Construction of risk model. (**A**) Minimal crossover validation points show the number of hub genes and the trajectory of each independent variable based on the training set. (**B**) Risk model identification in training and test cohorts.

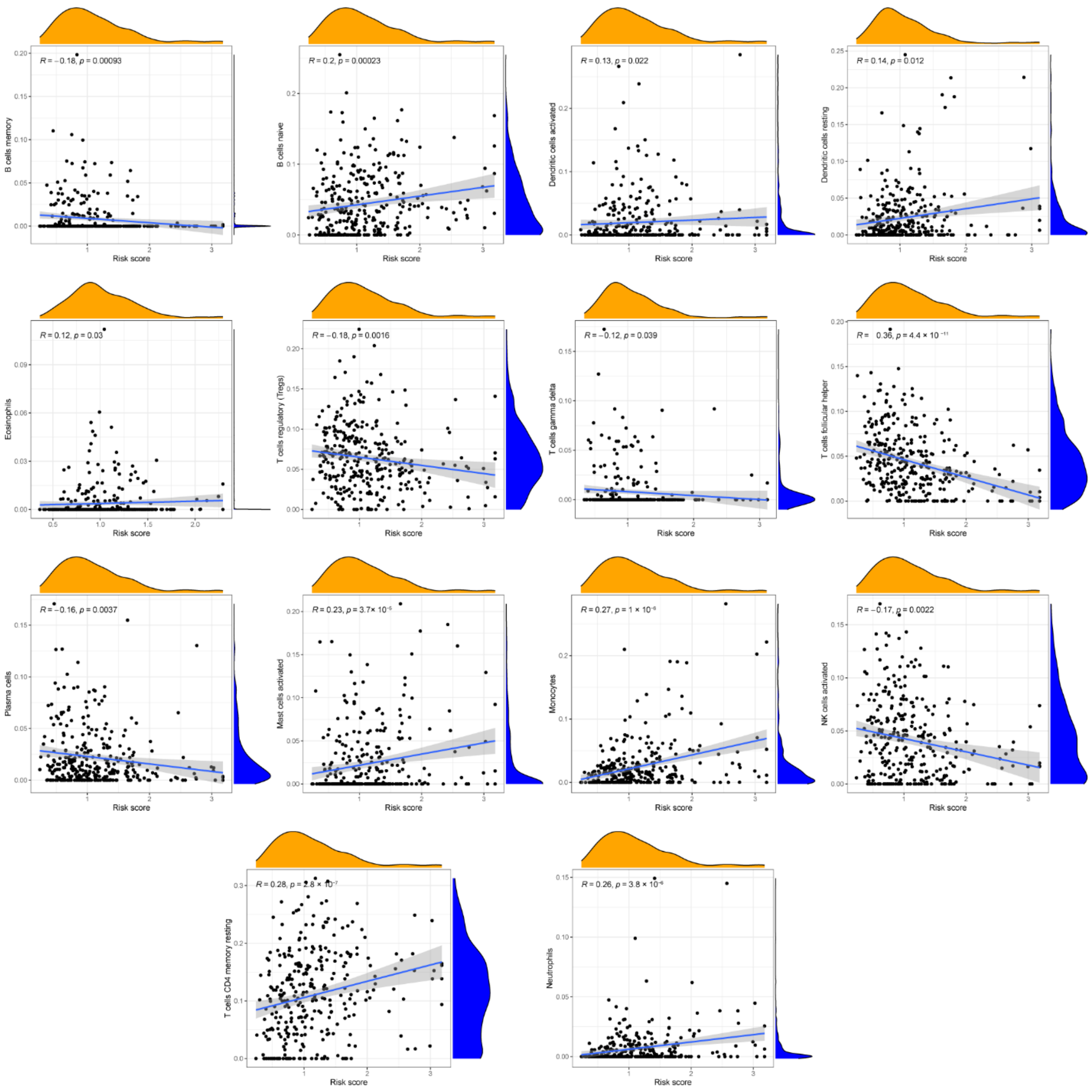

Supplementary Figure S6: The association between risk score and immune cells.
