# Supplementary figures and images for: Identification of a Novel Model for Predicting the Prognosis and Immune Response Based on Genes Related to Cuproptosis and Ferroptosis in Ovarian Cancer
Source: Cancers (Basel). 2023 Jan 18;15(3):579. doi: 10.3390/cancers15030579 (PMC9913847; doi:10.3390/cancers15030579)

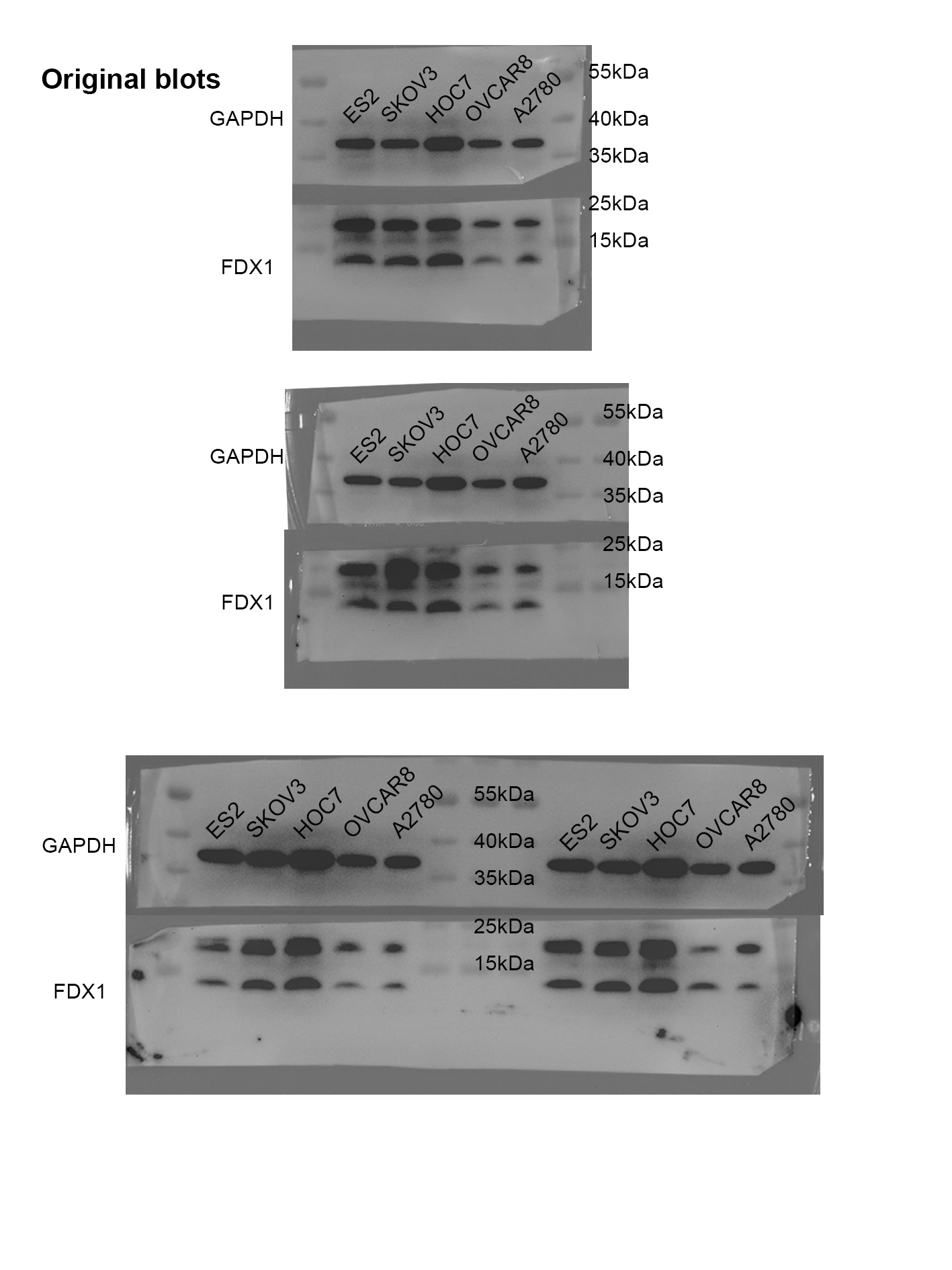

Supplement: Supplementary file 1 [file cancers-15-00579-s001.zip › Supplementary File S1.tif]
